# Factors Affecting the Impact of Off-Road Driving on Soils in an Area in the Kruger National Park, South Africa

**DOI:** 10.1007/s00267-012-9954-y

**Published:** 2012-10-16

**Authors:** Gerhardus Petrus Nortjé, Wouter van Hoven, Michiel C. Laker

**Affiliations:** 1 Centre for Wildlife Management, University of Pretoria, South Street Lynnwood Pretoria, Pretoria, Gauteng 0002 South Africa; 2 Centre for Wildlife Management, University of Pretoria, Pretoria, Gauteng South Africa

**Keywords:** Soil compaction, Off-road driving, Tyre pressure, Penetration resistance, Vehicle passes, Vehicular traffic

## Abstract

Studies on the effects of off-road driving on soils were conducted in the Makuleke Contractual Park of the Kruger National Park. The studies were conducted on three different soils with different textures and soil compactibilities. Traffic pressure was applied with a game drive vehicle loaded with 11 sand bags, each weighing 70 kg. This gave a total vehicle mass of 3,795 kg, simulating a vehicle fully laden with tourists. The study included: (i) comparing of the effects of four different tyre pressures; (ii) comparing the effects of 1–3 vehicle passes over the same tyre tracks; (iii) comparison of traffic effects under dry and wet soil moisture conditions, on soil compaction, respectively. After each pass penetration resistances were measured (a) on the tyre tracks, (b) between the tyre tracks and (c) at different distances outside the tyre tracks. As expected, vehicular traffic caused soil compaction below the wheel tracks. Lower tyre pressures caused less compaction than higher tyre pressures. Fewer vehicle passes also caused less compaction than more passes on the same tracks, but most compaction occurred during the first pass. Thus, driving on the same tracks more than once is less damaging than driving once on different tracks. Controlled traffic should be considered when developing management strategies for off-road driving in wildlife protected areas.

## Introduction

As part of the South African National Parks (SANParks) commercialization process in the Kruger National Park (KNP), concession areas were set aside for the exclusive use of private operators (Nortjé 2005). The objective of the commercialization process is to broaden the tourism product of the KNP and, thereby, increase the revenue for the SANParks (Nortjé 2005).

Concession operators are allowed certain tourist-attracting activities, including off-road driving (ORD), aimed at bringing tourists in close contact with members of the ‘Big Five’ in wildlife. It seems as if such activities are often implemented without knowledge regarding the full potential impacts of the activities on the environment and more particularly the soils (Nortjé 2005). Certain principles and guidelines were set for practising these activities in the concession areas, but some of these guidelines and principles have not been tested and/or not scientifically proven. ORD is a case in point.

One of the guidelines for ORD states that (Van der Merwe 2004): “Vehicles that drive off-road may not follow in each other’s tracks”. This is the practised guideline that is still being continued after several years. The objective of the research reported here was, thus, to determine whether vehicular off-road traffic impacts on soil compaction and if it does, to quantify the magnitude of the impact on soil compaction.

Soil compaction is defined as the process of bringing soil to a dense state, i.e. increasing its bulk density (Van der Watt and Van Rooyen 1995). Soil compaction can basically be distinguished as (i) soil crusting (formation of a seal at the soil surface) and (ii) subsurface compaction (the formation of a dense soil layer some distance below the soil surface). The latter is usually meant when the term “soil compaction” is used. Numerous studies on the effects of soil compaction on plant growth have been conducted since about the early 1960s, mainly in the USA, Australia and South Africa. These have been reviewed by, amongst others, Bennie and Krynauw (1985), Du Preez and others (1979, 1981) and SASTA (2001). The vast majority of these studies were conducted in croplands, both dry land and irrigated. The key factor is the effects of soil compaction on root penetration. The researchers came to the conclusion that bulk density was not the best factor to use in root penetration studies.

In addition it is quite cumbersome for routine determinations. It was found that “soil strength”, defined as “a general term referring to the ability of a soil to resist deformation by applied forces” (Van der Watt and Van Rooyen 1995) or the soil’s mechanical resistance to penetration by plant roots. The instrument used to measure this is a penetrometer, which measures “penetrometer resistance”. A thin metal probe is driven into the soil and the resistance of the soil to its penetration, i.e. the force required to drive it in, measured. In modern penetrometers the probes are driven in electrically at a constant rate and resistances determined and recorded electronically.

The effects of high soil compaction on plants include:

- Inability of roots to penetrate through the compacted layer and thus inability to utilise water stored in the subsoil. This makes plants much more vulnerable to drought stress, especially when dependent on low and erratic rainfall;
- Roots not only becoming shorter, but also thicker, thus having lower specific surfaces (less feeding surface per unit root mass). The consequence is very poor uptake of a whole range of essential plant nutrients, including especially phosphorus (Bennie and Laker 1975; Du Preez and others 1979, 1981; Merotto and Mundstock 1999). This leads to induced nutrient deficiencies and poor plant growth.

In addition to the reduction in soil productivity, soil compaction also increases erodibility, thus “affecting additional compartments in the surrounding ecosystems” (Horn and Fleige 2009). Soil compaction is mostly irreversible (Horn and Fleige 2009), meaning that the soil will not recover unless the compacted layer is broken up with tined implements, as used in crop farming.

Research in agriculture has established that vehicular traffic is the primary source of the mechanically applied forces to soils which lead to soil compaction, with concentrated pressure under the wheels being the greatest contributing factor (Bennie and Krynauw 1985). By far the biggest part of compaction (up to 90 %) takes place during the first pass of wheels over an area (SASTA 2001; Du Preez and others 1979, 1981). Subsequent wheel passes on the same tracks increase the degree of compaction under the tracks little compared with the first pass. Thus, uncontrolled haphazard movement of tractors, implements, harvesting machinery, lorries, etc., over cultivated fields during secondary operations can compact the whole field, causing the development of a sub-surface “traffic pan”. In contrast, Du Preez and others (1979, 1981) found that a simple cultivation system of controlled traffic greatly reduces the compacted area. Van der Watt and Van Rooyen (1995) define controlled traffic as: “Tillage in which all operations are performed in fixed paths so that re-compaction of soil by traffic (traction or transport) does not occur outside the selected paths”. Controlled traffic has been used by farmers in various parts of the world as an effective management technique to minimize soil compaction under intensive crop production systems for more than 50 years. It has also been practised very effectively by South African farmers for about that same period of time.

In the South African forestry industry it was also found that overall productivity decline depends on the areal extent of the harvesting operations and thus on the area compacted during harvesting (Smith and Johnston 2001). Smith and Johnston (2001) pointed out that 40 % growth loss over 10 % of an area is very small compared to 20 % growth loss over 80 % of the area. Bekker (1961) found that subsoil compaction caused by wheels is not confined to the area directly under the wheels. On both sides of a track compaction takes place at angles of 45° from the side of the track. Thus, the area compacted is much wider than the wheel track itself.

It was found that the degree of compaction (density of the traffic pan) is determined by the tyre pressure of a vehicle travelling over the soil (SASTA 2001). The higher the tyre pressure is the more severe is the compaction.

Each soil has a specific soil water content at which it is most susceptible to compaction when pressure is applied to it, for instance, by a tractor tyre. Numerous South African studies have been done on this in the agricultural and forestry sectors, as, for example, reported in several papers in SASTA (2001), Bennie (1972), Henning and others (1986). It is accepted that maximum compaction occurs at fairly high soil water contents—just below field capacity. Conditions under which ORD is done in game reserves are somewhat different from those in agriculture and forestry. The main difference is that in game reserves ORD is usually done on virgin, undisturbed soils—although this is not always the case. Thus the wheel impact of vehicles may be somewhat different than in agriculture and forestry. Some studies have been done elsewhere on impacts of ORD in game reserves, for example, by Bhandari (1998), Onyeanusi (1986), McCool (1981) and O’Brien (2002).

The latter studies mentioned above did not include basic measurements of the effects of ORD on soil physical conditions, such as sub-surface compaction. No clear guidelines and recommendations could, therefore, be derived from them. A comprehensive study was thus conducted regarding the potential impacts of ORD on soil conditions and consequently on plant growth. Some attention was given to recovery potential from the impacts of ORD. The perceptions of tourists were also studied. This paper reports on the impacts of ORD on soil compaction.

## Materials and Methods

### The Study Area

**Fig. 1:**
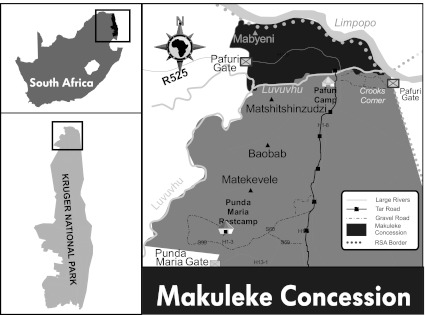
The Makuleke Contractual Park

Field experiments were initiated during March 2010 on three different sites in the Makuleke Contractual Park (MCP), in the Northern KNP, South Africa. The MCP is situated between the Limpopo and Luvuvhu Rivers in the northern sector of the KNP, South Africa (Fig. 1). This 24,000 hectare area is recognised as one of the most diverse and scenically attractive areas in the KNP and is called either the Pafuri triangle or the Makuleke Concession- as it is the ancestral home of the Makuleke people (Pafuri factsheet 2011).

The Makuleke area is the meeting point of a multitude of habitats, resulting in a region of incredibly rich biodiversity. The reasonably low annual rainfall of between 375 and 400 mm per year belies the fertility of the area which is by far the most diverse within the whole KNP with more than 70 % of the Park’s bird, mammal, fish, amphibian, reptile and tree species being found here (Pafuri factsheet 2011). The concession has mild winters from May to September with occasional chilly evenings, however summers are generally very warm.

The variety of habitats is also exceptionally scenic: from the pans and floodplains of the Limpopo and Luvuvhu Rivers to the cool riverine forests along their banks, rugged *kopjes* covered in mopane, giant baobabs and charismatic commiphoras, gorges carved from ancient rock, acacia-shaded savannah and the renowned fever tree forests. Many tree species reach the southernmost extremity of their ranges here. The MCP part of the Pafuri Land System consists of five landscapes according to Gertenbach (1983) namely: Punda Maria Sandveld on Cave Sandstone, *Adansonia digitata/Colophospermum mopane* Rugged Veld, *Colophospermum mopane* Shrubveld on Calcrete, Mixed *Combretum spp./Colophospermum mopane* Woodland and Limpopo-Luvuvhu Flood Plains.

### Selection of Trial Sites

**Fig. 2:**
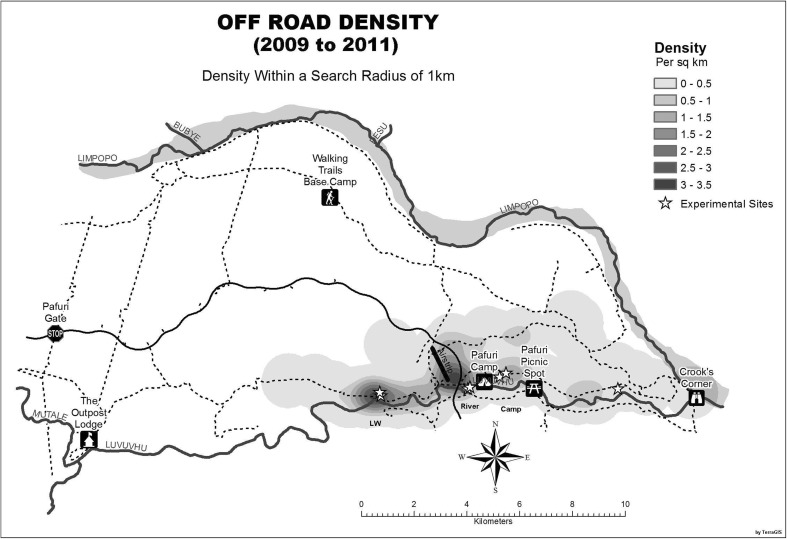
Map indicating the frequencies of ORD in the different areas showing the three trial sites

The trial sites were chosen by identifying the areas in which ORD occurred most and selecting a representative site in each of these (Fig. 2). This was conducted by analysing off-road data from previous animal sightings, for which ORD was approved. These sites were also selected after one year of practising ORD. They were selected to represent the most important soil types in the specific areas.

### Methods of Simulating ORD

The vehicle used to simulate ORD situations was a game drive vehicle with a roof rack, having a vehicle mass of 3,025 kg. It was loaded with 10 sand bags averaging 70 kg per bag, representing the maximum number of passengers, plus the driver/Guide. Thus the total mass came to 3,795 kg.

**Fig. 3:**
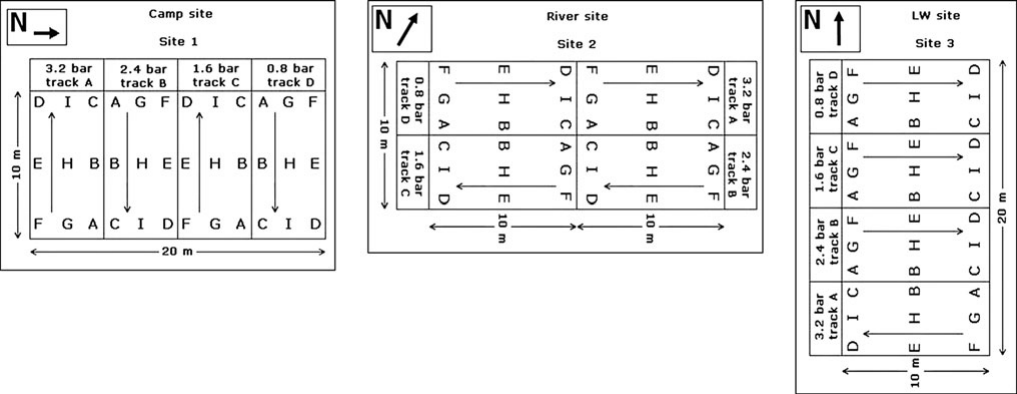
Soil compaction trial layouts

The vehicle had tyres 190 mm wide and inflated to 3.2, 2.4, 1.6 and 0.8 bars, equivalent to 320, 240, 160, and 80 kPa, respectively. The game drive vehicles operate at a tyre pressure of 2.4 bars or 240 kPa. The vehicle was driven across each trial site at a steady speed to produce sets of tracks which consisted of one, two and three vehicle passes. These passes were done for all tyre inflation pressures mentioned above and were 10 m in distance. A diagrammatic representation of the trials layouts is shown in Fig. 3 (for each tyre pressure the first pass of the vehicle was in the direction of the arrow for a distance of 10 m. The second pass was in reverse, and the third pass again in the direction of the arrow (the numbering letters, A to I, were used for statistical purposes and indicate control readings).

### Measurement of Soil Strength

A Geotron-P5 electronic penetrometer (Geotron Hand Penetrometer Model G 94), with a 30^°^ cone tip was used to determine the penetration resistance for each treatment. Each single treatment consisted of one tyre pressure, while driving over the same track, three times. Each treatment was conducted on a separate track. The penetration resistance, or soil strength, was measured at the following positions on and in between the tyre tracks: front/entrance (F G A), middle (E H B) and rear (D I C) of the tracks (Fig. 3).

A total of 12 measurements were taken for each treatment as follows: before the passing of the vehicle over the track (control measurements) and after each pass of the vehicle for a total of three passes at the same positions. These control measurements were taken on the vehicle tracks, in the middle between the tracks and at a specific distance outside of the vehicle tracks, also at the abovementioned, front, middle and rear positions. Thus the total number of measurements for each treatment was equal to 60. For a total of four tyre pressures this amount to a total of four times 60 = 240 readings per trial.

The compaction trials were conducted at two moisture regimes at each site. The dry condition trials were done during March 2010. Usually this is during the end of the rainy season, but in 2010 it was a dry period. The wet condition trials were done during April 2010 after good rains. Gravimetric soil water content was determined by taking representative top soil and sub-soil samples at different depths before each experiment commenced. This was conducted early in the morning for all three trials for consistency. The soil samples were weighed on an electronic scale and then microwave dried for up to 10 min whilst weighing at 1 min intervals until a constant mass was obtained. The soil water content values are given in Table 1. Soil water content is given as a mass percentage per mass oven-dry soil, i.e. (m_w_ × 100)/m_s_, as is convention in soil physics.

**Table 1:** Soil water contents

Soil depth (cm)	0–20	20–40	40–60	Field capacity (%)		Top soil pH
Site name	Average soil water (% dry mass)			Top soil	Sub soil	
Camp site (dry)	6.35	3.75	3.16	18.18	19.79	6.8
Camp site (wet)	14.25	9.76	7.47			
River site (dry)	3.31	3.76	4.32	14.80	8.34	6.8
River site (wet)	14.02	7.86	7.28			
LW site (dry)	7.56	6.73	5.35	19.13	21.09	6.4
LW site (wet)	13.89	10.18	8.03			

It has been found that the soil water tension at which water is held after all free water has drained from a soil differs widely between soils. Thus, the traditional approach of using the soil water content at 33 kPa soil water tension as indicator of so-called “field capacity” is no longer considered valid. Instead field determined field capacity, or the “drained upper limit” (DUL), is used as the upper limit of water held by a soil (e.g., Cassel and others 1983; Annandale and others 2011). Field water content in this trial was thus determined by wetting of the soil and allowing all free water to drain from the soil to a constant mass after 2–3 days.

One-way Analysis of Variance (ANOVA) was conducted to compare the average soil strengths across the number of passes at depths of 0–5, 6–15, 16–25 and 26–35 cm below the soil surface, for each trial site (α = 0.05). Multiple comparisons, Bonferroni correction, were performed post hoc to determine between which passes the statistically significant differences occurred.

### Characteristics and Properties of Soils at Trial Sites

Camp Site (Site 1) and LW Site (Site 3) are on soils of the Oakleaf form, and River Site (Site 2) on a soil of the Dundee form according to the South African soil classification system (Soil Classification Working Group 1991). The Oakleaf soils are classified as Cambisols according to WRB (1998) and the Dundee soils as Fluvisols. The soils of Sites 1 and 3 are typical Oakleaf soils, being pedogenetically young soils in early stages of development on a large sub-recent river terrace (the second terrace). There is a clay increase from the topsoil to the weakly structured subsoil. The Dundee soil of Site 2 is a typical soil with alluvial stratifications on the lowest terrace next to the river, presently being affected by sediment deposition by the river. There were important differences between the three soils regarding their chemical and physical properties and characteristics.

Particle size distribution (soil texture) is closely related to bulk density and is an important indicator of a soil’s susceptibility to compaction (Reed 1983). “It was established that of many factors that may influence soil compactibility, particle-size distribution is the most important for a group of soils studied” (Van der Watt 1969, p 79). The particle size distribution of the three trial sites differ substantially in respect to aspects that may affect soil compaction (Table 2).

**Table 2:** Particle size distribution

Site name	μm														
	1000 (%)	500 (%)	250 (%)	180 (%)	125 (%)	106 (%)	63 (%)	53 (%)	Pan (%)	0.05–0.02 (%)	0.02–0.002 (%)	<0.002 (%)	Texture	Sand (grade)	Compactability
Site 1 top (1T)	0.47	7.77	8.91	12.69	4.16	4.11	4.63	0.68	0.39	21.15	15.15	18.81	Lm	Fine	High
Site 1 sub (1S)	0.6	2.75	3.04	4.78	1.82	7.89	13.6	1.12	0.45	19.17	15.87	27.92	Lm-ClLm	Fine	Low-medium
Site 2 top (2T)	0.57	4.5	36.23	13.96	4.7	6.24	5.72	2.61	0.23	8.22	6.42	9.09	LmSa-SaLm	Fine	Very high
Site 2 sub (2S)	1.23	7.05	63.48	10.33	2.91	4.27	2.47	0.28	0.08	2.08	1.88	2.08	Sa	Fine	Very high
Site 3 top (3T)	0.41	0.12	0.34	1.1	4.29	13.8	19.13	10.07	0.88	20.32	9.62	19.48	Lm-SaLm-SaClLm	Medium	Medium–high
Site 3 sub (3S)	0.4	0.02	1.33	0.32	1.24	12.83	18.47	4.68	0.84	21.97	11.8	25.18	Lm	Medium	Medium–high

The Oakleaf soils at Sites 1 and 3 are similar in regard to:

- Clay content, including similar topsoil clay contents, similar subsoil clay contents and similar increases in clay content from topsoil to subsoil;
- Silt content, being high relative to the values for most South African soils, but common for Oakleaf soils.

These soils differ substantially in regard to their fine sand content, a very important factor regarding susceptibility to soil compaction (Laker 2001; Bennie and Burger 1988). The soil at Site 1, especially the topsoil (1T), has a much lower fine sand (< 100 μm) content (26.7 and 29.7 % for top- and subsoil (1S), respectively) than that for top- (3T) and subsoil (3S) at Site 3 (49.8 and 38.4 %, respectively). This means that the fine sand plus silt content of the soil at Site 1 is more than 60 % and at Site 3 more than 70 %, with the topsoil nearly 80 %. Serious compaction is normally expected in soils with more than 50 % fine sand plus silt, especially if silt is more than 20 %, and less than 35 % clay (Laker 2001). Expressed as a fraction of the sand content of the soils the fine sand proportions are about 60 % for the topsoil at Site 1 and 82 % for the subsoil, compared with more than 95 % for both the top- and subsoils at Site 3. The implications of these are discussed later.

In contrast to the others, the soil at Site 2 is a sandy soil. The subsoil (2S), with only 2 % clay and 3 % silt, is classified as having pure sand texture. The sand fraction is also much coarser than at the other two sites, being dominated by medium sand and with relatively little fine sand.

**Fig. 4:**
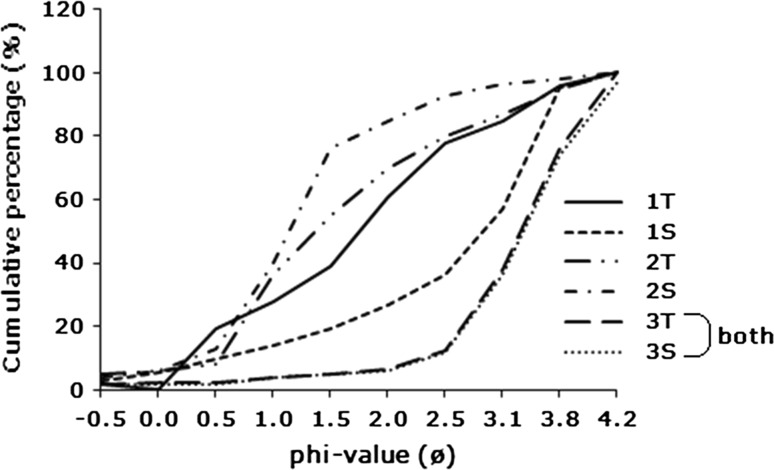
Cumulative phi-value curve (Laker 2011, pers comm)

The degree of sorting of the sand fraction of a soil is also a factor to consider. At Site 1 sorting in the sand fraction of both the topsoil and subsoil is poor, but close to moderate due to fairly sharp increases in parts of the cumulative phi value curves (Table 3; Fig. 4). At Site 3 sorting is moderately well, as indicated by sharp increases in cumulative curves between phi values of 2.5 and 3.8. At Site 2 the topsoil (2T) is very close to moderately sorted and the subsoil moderately well. Henning and others (1986) found that soils with moderately sorted sand fractions were more prone to soil compaction than soils with poorly sorted sand fractions. Moolman and Weber (1978) found extreme compaction of well-sorted fine sandy soils in the south-western cape of South Africa. They did not expect such well-sorted soil to be prone to compaction, but *“yet it happens”*. They expected that a well-graded soil, with a good mixture of different particle sizes would be a prerequisite for severe compaction. Bennie and Burger (1988) describe the majority of soils that are susceptible to compaction at Vaalharts as *“(…) characterised by a high fine sand fraction, low clay and organic matter content, single grain to weakly massive structure and particle size with good sorting.”* Thus, sorting of their sand fractions could contribute to making the soils at the trial sites more vulnerable to compaction, although it is evident that sorting alone does not give complete explanation for the vulnerability of soils to compaction.

**Table 3:** Sand fraction sorting (sorting, skewness and curtose)

Soil	phi value	Class
1T	1.25	Poor
1S	1.15	Poor
2T	1.02	Poor
2S	0.61	Moderately well
3T	0.62	Moderately well
3S	0.62	Moderately well

Relevant class limits	
Class	Class Limits
Moderately well sorted	0.50–0.70
Moderately sorted	0.70–1.00
Poorly sorted	1.00–2.00

Clay mineralogy plays an important role in determining the susceptibility of soil to disaggregation of aggregates, and thus also in its vulnerability to crusting and erosion (Stern 1990; Bühmann and others 1996; Rapp 1998). This would also be the case with vulnerability to compaction. Usually soils with clay fractions dominated by smectite are considered the most vulnerable to dispersion and disaggregation, while those dominated by kaolinite are considered to be quite stable (Rapp 1998). However, in South African studies, it has been found that soils in which kaolinite is dominant, but occurs in combination with significant amounts of smectite, are very vulnerable to disaggregation (Stern 1990; Bloem and Laker 1994). On this evidence the Oakleaf soils of Sites 1 and 3 should be highly prone to disaggregation and compaction (Table 4). It has been found that soils with high quartz contents in their clay fractions are found widespread in South Africa (Laker 2004). It has been found that soils with high quartz contents in their clay fractions are extremely prone to disaggregation, crusting and erosion (Bühmann and others 1996) and also to subsurface compaction (Moolman and Weber 1978). This would then be an important factor at especially Sites 1 and 2.

**Table 4:** Mineralogy clay analysis

6 treatments							
Site name	Quartz (Qz)	Smectite (St)	Kaolinite (Kt)	Mica (Mi)	Talc (Tc)	Feldspar (Fs)	Hematite (Hm)
Camp (1T,1S)	35	28	29	8	0	0	0
River (2T,2S)	41	13	10	22	5	9	0
LW (3T,3S)	15	30	41	11	1	0	2

In terms of chemical properties all the soils in this study have low organic matter contents (Table 5), which would increase their vulnerability to disaggregation and compaction. Relatively high exchangeable sodium contents or lopsided Mg:Ca ratios would also increase the vulnerability of soils to disaggregation (Bloem and Laker 1994), but these are not problems in the soils of the present study (Table 5).

**Table 5:** Soil chemical properties

Site name	pH (H_2_O)	Na	K	Ca	Mg	S-value	CEC	% C (top soil)
		cmol(+)/kg						
1T	6.20	0.33	0.40	6.65	4.05	11.43	13.91	1.12
1S	6.69	0.40	0.29	10.57	5.77	17.02	17.42	
2T	7.97	0.07	0.23	6.18	2.86	9.34	8.32	1.15
2S	8.10	0.02	0.06	2.23	1.27	3.58	2.59	
3T	6.91	0.47	0.46	7.49	4.21	12.63	13.33	1.06
3S	5.61	0.13	0.16	10.53	5.98	16.79	19.02	

It would thus seem that unfavourable particle size distribution and clay mineralogical composition of the soils in the study could be key factors aggravating their potential vulnerability to both crusting and subsurface compaction.

## Results and Discussion

### Penetrometer Resistance Results

**Fig. 5:**
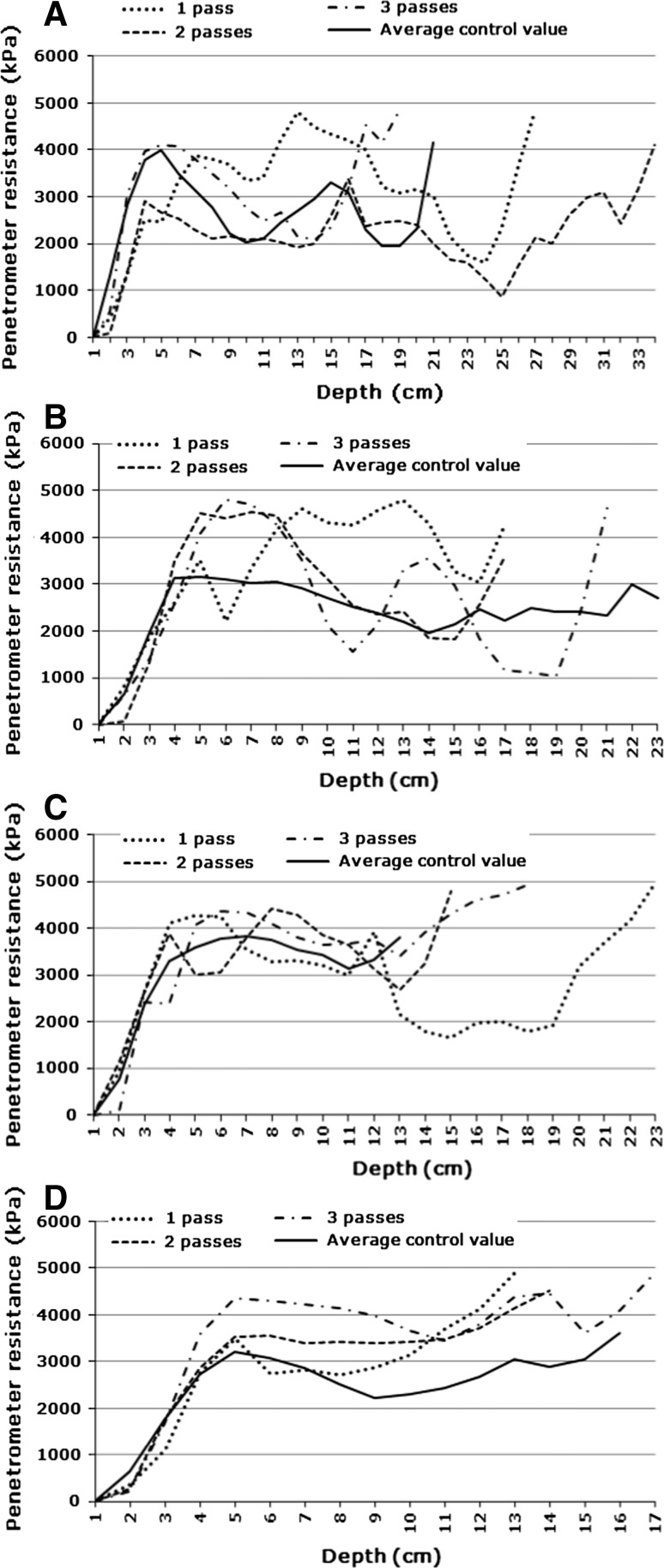
Average control value vs. track values at **a** 0.8 bar and 1–3 passes (Site 1, dry); **b** 1.6 bar and 1–3 passes (Site 1, dry); **c** 2.4 bar and 1–3 passes (Site 1, wet); **d** 3.2 bar and 1–3 passes (Site 1, wet)

**Fig. 6:**
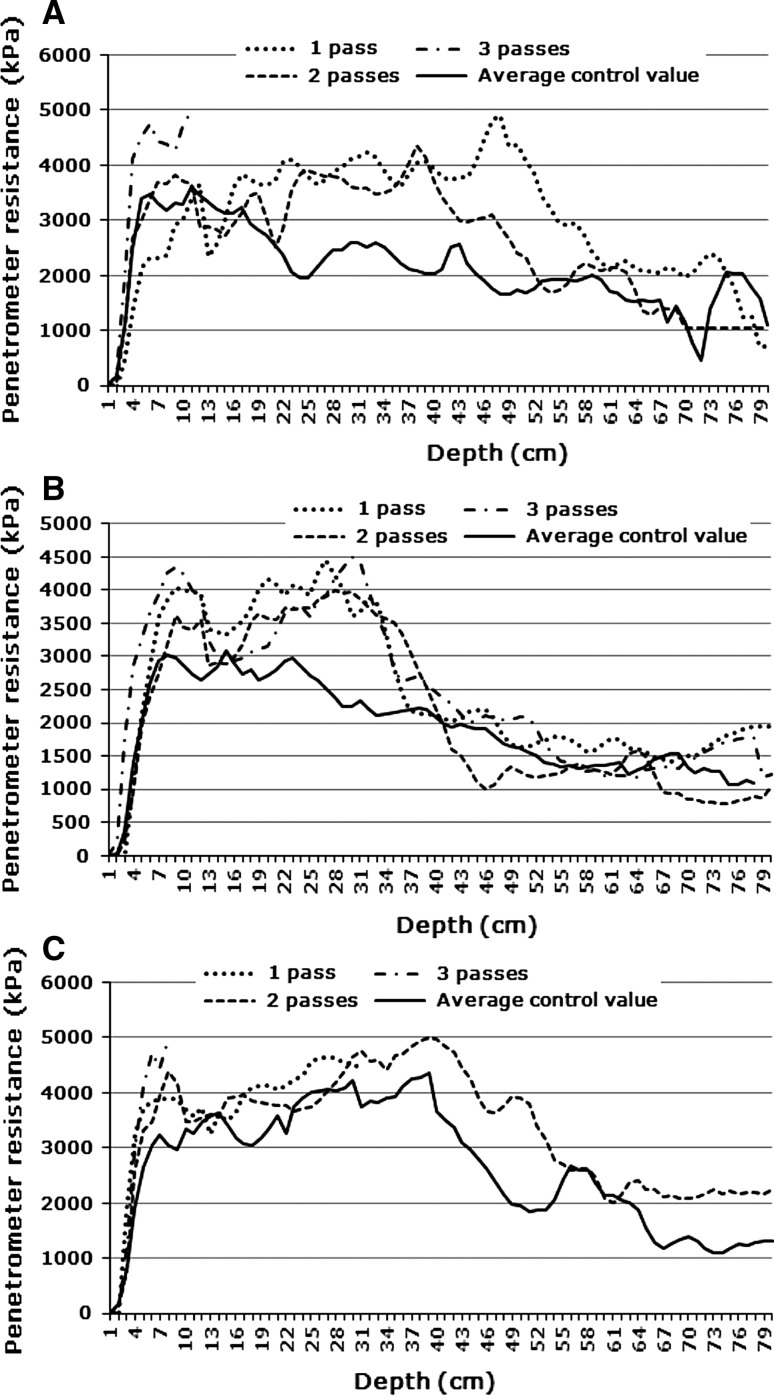
Average control value vs. Track values at **a** 0.8 bar and 1–3 passes (Site 2, wet); **b** 1.6 bar and 1–3 passes (Site 2, wet); **c** 3.2 bar and 1–3 passes (Site 2, wet)

**Fig. 7:**
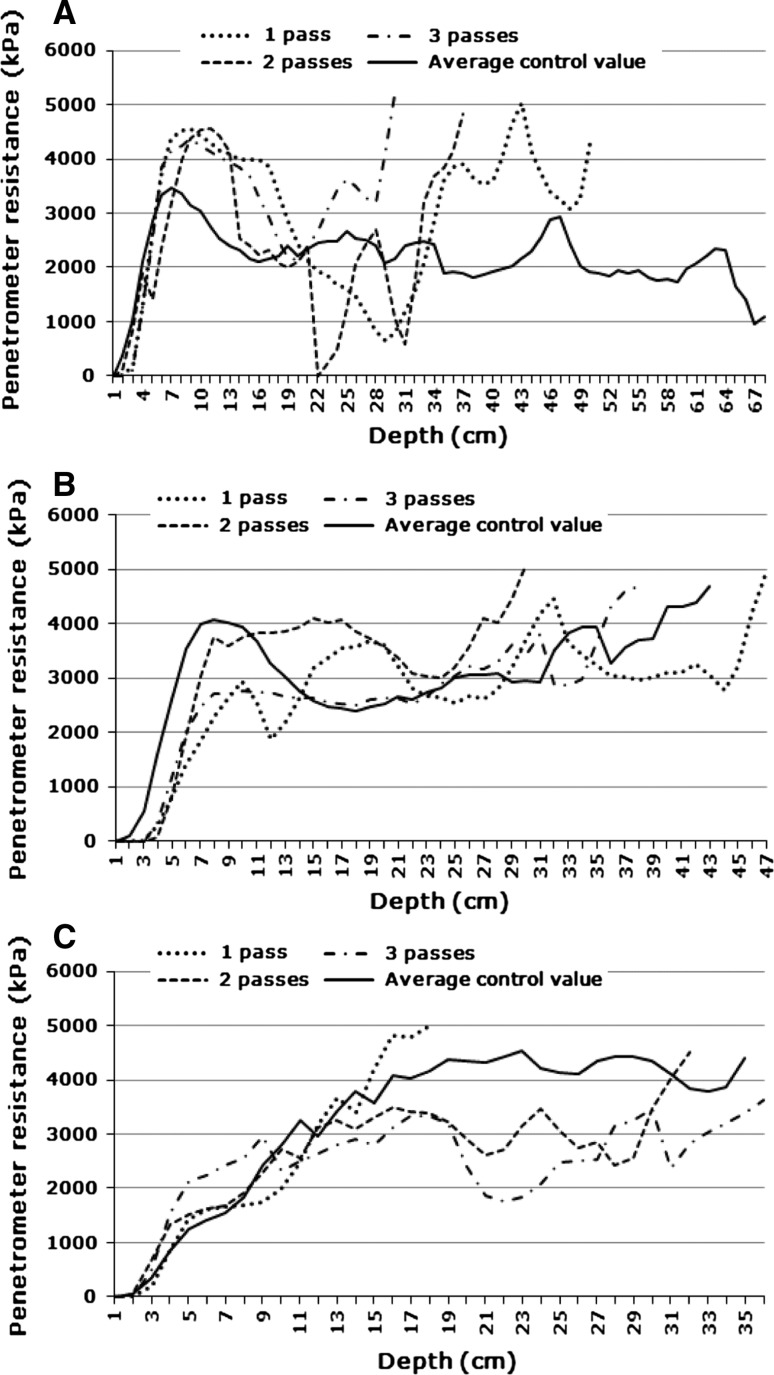
Average control value vs. Track values at **a** 0.8 bar and 1–3 passes (Site 3, dry); **b** 1.6 bar and 1–3 passes (Site 3, dry); **c** 2.4 bar and 1–3 passes (Site 3, wet)

Penetrometer resistance (soil strength) results are presented in Figs. 5, 6 and 7. These are only for the cases where statically significant differences were found. Differences were found in all cases but several were not statistically significant.

It will be noted that in all cases soil strength values start at very low values at the soil surface and then increases with depth to a fairly shallow depth. This is an artefact of the penetrometer measuring technique. Because of the cone shaped tip, soil is pushed up around it to the unconfined soil surface. Visual inspection revealed that in most cases these soils had dense crusts (surface seals). Penetrometers cannot be used to detect or measure surface crusts. In the present study this is not relevant, because the study aimed at determining subsurface compaction only.

Some authors consider a soil strength of 2,500 kPa as the threshold value above which root growth becomes restricted (e.g., Greacen and Sands 1980; Laker 1987), while others consider 2,000 kPa to be the threshold (e.g., Adams and others 1982; Van Huysteen 1983; Bengough and others 2011). This lower soil strength threshold value of 2,000 kPa seems to be more generally accepted presently (Van Antwerpen 2011, pers comm) and was therefore chosen for this study.

### Penetrometer Resistances of Controls

The penetrometer resistance values of the control measurements were high throughout (Figs. 5, 6, 7). Naturally occurring dense subsoils are not uncommon in South Africa (Bennie 1972). It was also found in the Eastern Cape for Oakleaf soils with textures very similar to those at Sites 2 and 3 of the present study (Du Preez and Botha 1980) in a region where quartz in the clay fraction is common.

In some cases at Sites 1 and 3 there are distinct very high soil strength values close to the soil surface. It was later found that the Makuleke people cultivated these areas up to 1969, when they were removed (Pafuri factsheet 2011). This resulted in severe crusting of the soils. Some large areas were still, after 42 years, barren and devoid of any vegetation, showing the very poor resilience (recovery potential) of these soils. Webb (2002) found similar results in the Mojave Desert in California. The trial sites were not on such extreme areas. Sub-surface compaction did not occur, because ploughing was conducted by animal-drawn implements and other operations by hand cultivation with hoes. No mechanised implements were employed and thus no traffic pans could develop.

### Effects of Vehicular Traffic on Penetrometer Resistances

Vehicular traffic affected penetrometer resistances of the soil at all three sites, at all tyre pressures under both dry and wet conditions. Most of the differences were not statistically significant, though. It must be kept in mind that one is dealing here with a natural system with high spatial variability even over short distances due to, *inter alia,* effects of old root channels, termites, etc.

#### Site 1

Under dry conditions at Site 1 statistically significant differences occurred only at low tyre pressures (0.8 and 1.6 bar). The outstanding features at 0.8 bar (Fig. 5a) are:

- The major increase in penetrometer resistance, compared with the control, over the soil depth from 7 to 20 cm due to the first pass of the vehicle. Under mechanised cropping conditions there is normally a loose soil layer from 5 to 15 cm due to secondary cultivation and a very dense and severely restrictive traffic pan from 15 to 25 cm depth, about the same thickness as the one here (Bennie 1972). The much shallower occurrence of the compacted layer here has major implications in regard to root development and water availability;
- At 25 cm the first pass caused a very sharp increase in penetrometer resistance, indicating the top of a second severely compacted layer, similar to what Bennie (1972) indicated at the same depth;
- After the second pass the penetrometer resistance decreased to similar values as for the control. It could be due to cracking of the massive layer caused by the first pass, according to the mechanism described by (Braunack 1986a, b);
- During the third pass there was significant re-compaction near the soil surface and clear indication of the top of a severely compacted layer at 15 cm depth. This is about the depth where one normally finds plough layer compaction under cropping conditions (Bennie 1972), that is, the forming of a compacted layer in the bottom part of a loose plough layer. In this case in the relatively loose soil layer formed by the second pass.

At 1.6 bar tyre pressure there were certain similarities with the patterns at 0.8 bar tyre pressure (Fig. 5b), including:

- Severe compaction of the layer between a soil depth of about 7 and 17 cm by the first pass;
- Lowering of the penetrometer resistance in the bottom part of this layer during the second pass;
- Re-compaction in the latter relatively loose layer during the third pass;
- Clear indications of the development of severely compacted layers deeper in the profile after all three passes. The difference is that the top of this layer after the third pass was much deeper in the profile than at 0.8 bar tyre pressure.

A slight difference in this case is the serious compaction close to the soil surface (crust formation) after two and three passes, although not very different from the pattern after three passes at 0.8 bar.

Under wet conditions at Site 1 statistically significant differences occurred only at high tyre pressures (2.4 and 3.2 bar). The outstanding features at 2.4 bar (Fig. 5c) are:

- The control values were throughout high and over most of the depth to which there are control values there were no significant effects of vehicular traffic. Like under dry conditions there was a depth where the first pass increased soil strength, the second pass lowered it drastically and the third pass re-compacted it to the same value as after the first pass. This was at a very shallow depth (about 3–7 cm), in other words, a dense crust;
- At greater depth, beyond where there are control values, the soils had quite low penetrometer resistance values after the first pass, which was drastically increased by the second pass. So, it seems that there is a pattern that the first pass over soil with a relatively low penetrometer resistance is the really damaging one.

At 3.2 bar tyre pressure under wet conditions the first pass started giving higher values than the control only at about 9 cm depth. Only at about 11 cm this became a clear increase and joined the values for the second and third passes (Fig. 5d). From this depth downward in the profile the values for the three passes joined and were clearly much higher than the control, that is, the first pass was the damaging one. At very shallow depth (in the zone of a crust) the third pass was clearly the damaging one.

Under wet conditions the development of a crust due to vehicular traffic is the over-riding consequence of ORD on this soil. Crusting has serious long lasting effects like inhibiting root growth (Laker and Vannache 2001), germination and seedling emergence, the latter especially of small-seeded plants like grasses. Thus, wetlands should be absolutely prohibited areas as far as ORD is concerned, particularly at the normal tyre pressures used.

#### Site 2

At Site 2, the very sandy soil, statistically significant differences were found only under wet conditions. In the plots of the 0.8 bar measurements (Fig. 6a) the mean penetrometer resistance values in the top part of the profile, to about 20 cm, were very high, before decreasing to values at or just above the threshold value down to about 45 cm. From there downwards it drops to below the threshold value. The main impacts of vehicular traffic were:

- Down to about 10 cm depth the first pass lowered the soil strength, which then became re-compacted to its original value by the second pass and further seriously compacted by the third pass. Again a pattern of a dense layer broken up and then re-compacted. No readings could be taken deeper for the third pass because at 5,000 kPa the penetrometer cuts out as safety measure. Again, serious crusting is a major issue when driving over a wet soil;
- Between about 15 and 60 cm soil depth the first pass caused serious compaction of this relatively loose soil (compared with that at Site 1). Down to about 40 cm the values for the second pass more-or-less follow those for the first pass, thereafter dropping below them, down to about 60 cm, from where traffic had no further impact and the lines for the two passes joined that of the control. Normally one would not expect an impact to such depth, but this is an extremely sandy soil dominated by medium sand.

At 1.6 bar tyre pressure the pattern was much the same as at 0.8 bar, with just some depth differences (Fig. 6b). The main impacts were:

- Compaction at a shallow depth (around 10 cm) by the first pass, followed by lowering of the soil strength by the second pass and re-compaction by the third pass;
- Serious compaction by the first pass, with no further compaction by the subsequent passes, as shown by the lines for the three passes running together. From about 37 cm deeper the vehicular passes had no effect, as shown by all four lines, including the control, running closely together.

At 3.2 bar tyre pressure the most outstanding feature is again serious compaction near the soil surface (around 10 cm) by vehicular traffic under wet conditions — increasing with increasing number of passes (Fig. 6c). Again the measurement for the third pass stopped at shallow depth because a value of 5,000 kPa was reached. Deeper in the soil the first and second passes had little effect because the control already had very high soil strength values.

Thus, the findings for Site 2 strongly support those for Site 1 that vehicular traffic brings about severe crusting under wet conditions and that wetlands should clearly be declared prohibited areas in regard to ORD. On this sandy soil a much stronger crust formed than in the medium-textured soil at Site 1. On this very sandy soil serious subsurface compaction was also found due to vehicular traffic under moist conditions.

#### Site 3

Under dry conditions at Site 3 vehicular traffic caused significant differences in soil strength at low tyre pressures (0.8 and 1.6 bar), as was found in the similar soil at Site 1. The main findings at a tyre pressure of 0.8 bar were (Fig. 7a):

- The control soil in these plots had near-surface compaction (crusting) at a depth of between about 5 and 11 cm. The first pass caused a big increase in the penetrometer resistance of this layer and made it much thicker, covering a depth from 5 to 20 cm. The second and third passes did not bring about any further increases in the compaction;
- From about 20 to 35 cm depth the first pass reduced the soil strength below that of the control. The central part of this, where the biggest reduction took place, was re-compacted by the second pass;
- From about 30 cm depth there were very sharp increases in penetrometer resistance values over very short distances, indicating the top of a compacted layer, after both the first and second passes. After the third pass this feature shifted to a shallower depth. This is similar to what was found in the similar soil at Site 1 with the same tyre pressure.

The plots at a tyre pressure of 1.6 bar showed a similar compaction at a shallow depth around 10 cm (Fig. 7b). Main affects of vehicular traffic in this case were:

- At this higher tyre pressure the first pass broke up the compact layer, which was then re-compacted by the second pass and broken up again by the first pass. This fits in with findings at the other sites;
- Below this layer the first pass brought about some compaction and the third pass more, which was then actually broken up by the third pass.

Under wet conditions at Site 3 differences were found only at 2.4 bar tyre pressure and these were quite abnormal (Fig. 7c). There was no sign of near-surface compaction in the control. Penetrometer resistances of the topsoil were actually quite low. The first pass of the vehicle had no effect to a depth of about 15 cm below which there was a fairly sharp increase in penetrometer resistance above the control until it cut out at 5,000 kPa. The second and third passes then broke this up and produced significantly lower penetrometer resistances than the (quite dense) control and the first pass. The presence of termite activity in this area could be a complicating factor affecting the results. The differences are more extreme, but probably not completely different from trends found under wet conditions at the other sites.

## Conclusion

The most important finding of this study is that ORD has strong negative impacts on soil crusting and sub-soil compaction. An important finding is that these negative impacts are during both dry and wet soil conditions. The negative impact of ORD on soil compaction has, thus, much wider impacts, such as decreasing water infiltration and availability, limited root penetration, less vegetation cover and reduced recovery of soil compaction (resilience) and vegetation as clearly indicated in this and other studies (Bhandari 1998; Adams and others 1982; Knapp 1992). The overall conclusion that can be made from this study is that the passage of game drive vehicles damages surface soil structure, which lead to soil crust formation and sub-surface compaction.

A highly significant result is that most crusting and sub-soil compaction occurred during the first pass of the game drive vehicle. This proves that controlled traffic of off-road vehicles is the best option in this specific case. Controlled traffic is very important to minimize compaction, as for instance, pointed out in SASTA (2001). Driving in the same tracks during all off-road incidents does not significantly affect the degree of compaction under the tracks, but greatly reduces the compacted area (Laker 2001).

Another important finding is the role that historical human activities play in such study areas and how it may influence results. The results in this study are aggravated by the historical human activities in this study area, as indicated. These historical activities were the main cause of the surface crusting, and the resultant low vegetation growth in the area. This, therefore, explains partially the relatively high control values and also the soil’s higher susceptibility to compaction due to vehicle ORD.

Although the results are variable, the tendencies are that sub-soil compaction occurs at lower soil depths with lower tyre pressures, and deeper with higher tyre pressures. In the agricultural industry with loose soils, up to 70 % of sub-soil compaction occurs with the first pass, but under more natural conditions as in this trial, the first pass generated lower (10–46 %) of the total sub-soil compaction.

Vehicular traffic brings about severe crusting under wet conditions for both Sites 1 and 2. On the sandy soil of Site 2 a much stronger crust formed than in the medium-textured soil at Site 1. On the very sandy soil of Site 2, serious subsurface compaction was also found due to vehicular traffic under wet conditions.

The results indicate that a small number of passes with a medium size vehicle (total weight = 3,795 kg) was able to compact the soil to a considerable depth below the soil surface during all tyre pressures and all passes in dry and wet soil. In the absence of ameliorative measures, the compaction is likely to remain for very long (Webb and others 1986 and Knapp 1992). The soil strength values after vehicle passage were consistently above the threshold of 2,000 kPa for all trial sites and during all three vehicle passes at shallow (0–15 cm) as well as at deeper soil depths (25–35 cm).

The results also indicate that during dry soil conditions soil strength can be reduced by vehicular traffic (as during the second pass in some cases in this study). Braunack (1986a, b) found similar results.
